# Feline respiratory disease complex: insights into the role of viral and bacterial co-infections

**DOI:** 10.3389/fmicb.2024.1455453

**Published:** 2024-09-03

**Authors:** Grazieli Maboni, Sunoh Che, Rebecca Tallmadge, Eliana De Luca, Laura B. Goodman, J. Scott Weese, Susan Sanchez

**Affiliations:** ^1^Athens Veterinary Diagnostic Laboratory, Department of Infectious Diseases, College of Veterinary Medicine, University of Georgia, Athens, GA, United States; ^2^Department of Animal and Avian Sciences, University of Maryland, College Park, MD, United States; ^3^College of Veterinary Medicine, Cornell University, Ithaca, NY, United States; ^4^Department of Pathobiology, Ontario Veterinary College, University of Guelph, Guelph, ON, Canada; ^5^Department of Infectious Diseases, College of Veterinary Medicine, University of Georgia, Athens, GA, United States

**Keywords:** respiratory disease, cats, co-infections, diagnostic panels, statistical modeling analysis

## Abstract

Feline respiratory disease complex (FRDC) is a highly prevalent syndrome in cats that often result in fatal outcomes. FRDC etiology is complex, and often, multiple viral and bacterial pathogens are simultaneously associated with disease causation. There is limited information about the role of co-infections in pathogenesis and the current prevalence of pathogens in North America. We aimed to conduct a study using technical advances in molecular diagnosis and statistical modeling analysis to elucidate the occurrence of pathogens and how co-infections affect disease severity. We attained information from three diagnostic laboratories in North America regarding the occurrence of *Bordetella bronchiseptica*, *Chlamydia felis*, *Mycoplasma*, *Felid alphaherpesvirus* 1 (FeHV-1), feline calicivirus (FCV), and influenza A, along with age, seasonality, sex, and clinical signs. We also evaluated the role of co-infections in disease severity. These pathogens were also investigated in clinically normal cats (control). The most detected pathogens were *Mycoplasma*, FCV, and FeHV-1. Most pathogens were detected in the control group, highlighting the challenge of interpreting positive testing results. Co-infections of *Mycoplasma* and FCV, as well as *Mycoplasma* and FeHV-1, were important predictors of disease severity. Age, sex, and season had a minor impact on pathogen occurrence. This study provides new insights into FRDC and underlines the relevance of diagnostic panels to screen for a range of pathogens, providing knowledge for timely diagnosis and therapeutic interventions.

## 1 Introduction

Feline respiratory disease complex (FRDC) is multifactorial with high prevalence worldwide, especially in cats living in shelters or in any situation where there is close contact with other cats (Cannon, 2023; Litster, 2021; Cohn, 2011). The disease is an important cause of euthanasia in shelters after overcrowding (Bannasch and Foley, 2005) and can lead to increased and/or inappropriate use of antimicrobials, higher cost of medical care, and the possibility that affected cats will not be adopted (Cannon, 2023; Litster, 2021; Bannasch and Foley, 2005).

Multiple viruses, bacterial pathogens, and host and environmental factors can cause and maintain FRDC. Evidence shows that pathogen co-infections often play a simultaneous role in disease exacerbation (Litster, 2021; McManus et al., 2014). The most commonly reported primary pathogens causing clinical signs include feline herpesvirus-1 (FeHV-1), feline calicivirus (FCV), *Bordetella bronchiseptica*, *Chlamydia felis*, and *Mycoplasma felis* (Cannon, 2023; Litster, 2021; Cohn, 2011; Bannasch and Foley, 2005; Michael et al., 2021). These primary bacterial pathogens have been shown by 16S rRNA gene sequencing to be present in the nasal communities during acute infection (Dorn et al., 2017); however, they have also been commonly detected in clinically normal cats (Bannasch and Foley, 2005; Lee-Fowler, 2014; Berger et al., 2015). Influenza A virus infection causes respiratory signs in most animals, even though it is rare in the feline population worldwide. However, an outbreak of H7N2 virus (low pathogenic avian influenza) affected hundreds of cats in the USA in 2016 (Palombieri et al., 2022). There are also reports of occasional influenza cases in cats caused by other influenza A viruses (Palombieri et al., 2022). Secondary bacterial infection with *Pasteurella* spp., *Escherichia coli*, *Staphylococcus* spp., *Streptococcus canis,* and *S. equi* subsp. *zooepidemicus*, or *Micrococcus* spp. has been reported based on culture methods (Litster, 2021; Schulz et al., 2006). Further, *Moraxella, Bradyrhizobiaceae*, *Staphylococcus*, *Pasteurella*, *Chlamydia,* and *Streptococcus* were frequently observed in cats with FRDC based on 16S rRNA gene sequencing (Dorn et al., 2017), but the clinical relevance of these is unknown and maybe limited.

Typical clinical signs associated with most of the primary pathogens of FRDC include serous to mucopurulent ocular and/or nasal discharge, sneezing, coughing, conjunctivitis, and submandibular lymphadenopathy (Litster, 2021; Palombieri et al., 2022; Fernandez et al., 2017). More severe clinical signs can occur, including anorexia, dehydration, inappetence, fever, and dyspnea (Radford et al., 2009; Thiry et al., 2009). Signs of erosion and ulceration of the mucosal surfaces are generally associated with FCV and FeHV-1 infection (Palombieri et al., 2022), and severe systemic signs involving the lower respiratory tract, such as pneumonia, anorexia, depression and fever, can cause severe, including fatal, disease, especially in young animals (Slaviero et al., 2021). *M. felis* is commonly isolated from conjunctival and nasal swabs in healthy cats as well as cats with clinical signs of upper or lower respiratory tract infection. Still, *M. felis* has been reported to be associated with disease in both upper and lower respiratory tract (Le Boedec, 2017). *B. bronchiseptica* has been detected in healthy cats and those with mild signs; however, severe pneumonia can occasionally occur (Cohn, 2011; Egberink et al., 2009). Long-term or chronic infection with recurrent appearance of clinical signs is common, but not all cats develop chronic infection. It will depend on the bacterial strain or viral biotype, host age, host immunity, vaccination status, and the presence of concurrent disease. These factors also play a role in the disease outcome. Chronic disease course is especially common in FeHV-1 infection due to the viral latent infection (Litster, 2021; Thiry et al., 2009).

The overlap in clinical signs between infectious agents can pose challenges for diagnosing specific pathogens. Infection with a primary pathogen may precede a secondary change in the underlying microbiome structure due to the alteration of protective defense mechanisms and disruption of the underlying microbial community composition. This may allow other agents to infect the respiratory tissues (Dorn et al., 2017; Arnold et al., 2022). The presence of co-infections may lead to more severe clinical signs compared with single pathogen infections; however, the role of co-infections in FRDC remains unclear. Further gaps in the literature include the lack of recent epidemiological studies of respiratory pathogens’ prevalence in North America. Understanding the extent of disease occurrence facilitates improving or establishing new diagnostic assays, vaccination programs, and alternative treatments.

This study conducted an etiologic and epidemiologic investigation of the most common pathogens known to be involved in FRDC using samples from clinically affected and clinically normal cats that were received at three different diagnostic laboratories in North America. This study had two objectives: (1) to investigate pathogen occurrence according to age, seasonality, sex, and clinical signs, and (2) to evaluate the role of co-infections in the severity of clinical presentation. Understanding the disease prevalence and the effect of co-infections on the disease severity will allow the improvement or the establishment of new vaccination programs and alternative treatments and may aid clinicians in rapidly interpreting laboratory results and approaching challenging co-infection cases.

## 2 Materials and methods

### 2.1 Study population

This was a retrospective study of laboratory data from regular sample submissions to three different laboratories in North America, including the states of Georgia (laboratory A), New York (laboratory B), in the United States, and in the province of Ontario, Canada (laboratory C). Inclusion criteria comprised samples from respiratory specimens from domestic cats submitted to the participating diagnostic laboratories for the detection of feline respiratory pathogens between 2011 and 2020. Most cases had a case date, age, sex, accession number, assay code, assay name, and result (positive or negative). Clinical signs were provided for most cases except for those from Laboratory B. Incomplete data were identified as unknown for the purpose of this study.

Some combination of conjunctival, nasal, oropharyngeal, eye swabs, transtracheal washes, or lung tissues had been submitted (Supplementary Table S1) for each patient for detection of the following common feline respiratory pathogens: *Bordetella bronchiseptica*, *Chlamydia felis*, *Mycoplasma*, FeHV-1, FCV, and influenza A virus. Results were sent to us as de-identified data, and no additional clinical information was gathered.

To investigate the presence of pathogens in cats without clinical signs of respiratory disease (control group), nasal swabs were collected 4 h to 24 h postmortem from clinically normal cats (*n* = 51) that had been submitted to Laboratory A for post-mortem evaluation. Inclusion criteria of these control animals were based on the absence of a history of respiratory clinical disease according to the submitting veterinarian, and the absence of any post-mortem signs of respiratory disease. A board-certified pathologist performed complete postmortem and histological examinations to confirm that the animals were not affected by respiratory disease at the time of death. Cats with any macroscopic or histological lesion associated with respiratory diseases were excluded from the control group.

The information collected in this retrospective study was part of routine diagnostic procedures. Hence, it did not require Institutional Animal Care and Use Committee approval. Animal ID and client information were kept confidential.

### 2.2 Pathogen detection methods

Nucleic acid extraction from clinical samples submitted to laboratory A was performed as previously reported (Maboni et al., 2019) and analyzed by a quantitative PCR panel targeting *Bordetella bronchiseptica* (Hozbor et al., 1999), *Chlamydia felis* (Helps et al., 2003), *Mycoplasma* spp. (Chalker et al., 2004), FeHV-1 (Helps et al., 2003), and reverse-transcription quantitative PCR (RT-qPCR) for FCV (Meli et al., 2018) and influenza A virus (Spackman et al., 2003). At laboratory A, positive samples for *Mycoplasma* spp. were sequenced by the Sanger method for *M. felis* identification as previously described (Stimmelmayr et al., 2018). At laboratory B, nucleic acid extraction was performed as previously reported (Tallmadge et al., 2020), and quantitative PCR was performed for *Bordetella bronchiseptica* (Helps et al., 2005), *Chlamydia felis* (Hewinson et al., 1997), *Mycoplasma felis* (Söderlund et al., 2011), and RT-qPCR for influenza A virus (Shu et al., 2011). Detection of FeHV-1 and FCV was performed by virus isolation on cell culture. All PCR assays were performed as previously described in the provided references. At laboratory C, nucleic acids were extracted using the MagMAX-96 Viral RNA isolation kit (Thermo Fisher), and quantitative PCR was performed for FeHV-1 and RT-qPCR for FCV based on assays developed *in-house* (data not published).

### 2.3 Predictor and outcome variables

Information about age, sex, dates (seasonality), and clinical signs was obtained from the original sample submission form provided by the submitting veterinarian. The occurrence of pathogen by age was evaluated in four categories defined as kitten (1- to <7-month-old, coded as 1), junior (7 month to <3-year-old, coded as 2), adult (3- ≤11-year-old, coded as 3) and senior (>11-year-old, coded as 4). To investigate whether seasonality impacts the occurrence of pathogens, data were divided into cold and warm seasons as previously described (Maboni et al., 2019). The cold season was defined as October 15th to April 15th; the warm season was defined as April 16th to October 14th.

To assess the effect of the severity of clinical signs on the occurrence of pathogens, we categorized information regarding clinical signs of infectious respiratory diseases from the data obtained from the submission forms from laboratories A and C. Diseased cats were categorized according to severity of clinical signs: clinical score 1 (upper respiratory mild disease: coughing, sneezing, conjunctivitis, or nasal/eye discharge), clinical score 2 (upper respiratory severe disease: the same signs as clinical score 1 plus ulcers in the mouth, lethargy, depression, inappetence or fever), clinical score 3 (lower respiratory disease: pneumonia followed by one or more signs such as dyspnea, lethargy, depression, inappetence or fever) (Table 1).

**Table 1:** Clinical scores of respiratory signs from cats at the time of sample collection.

Clinical score	Clinical signs	Total number of cats*
0 (clinically unaffected)	No respiratory signs and no post-mortem findings	51
1 (upper mild)	Coughing, sneezing, conjunctivitis, or nasal/eye discharge	111
2 (upper severe)	Coughing, sneezing, conjunctivitis, nasal/eye discharge, in addition to ulcers in the mouth, lethargy, depression, inappetence or fever	32
3 (lower, pneumonia)	Pneumonia followed by one or more signs such as dyspnea, lethargy, depression, inappetence or fever	45

*Clinical signs for 316 cases were unknown.

### 2.4 Statistical analysis

Differences in the proportion of pathogens across season, sex, clinical sign categories, and age were assessed by Fisher’s Exact test using Holm post-hoc test to adjust for multiple comparisons using R 4.3.0 software. *p*-values < 0.05 were considered significant.

A co-infection was categorized as 0 when there was no infection or infection with one pathogen, whereas it was categorized as 1 when cats were infected with more than one pathogen.

To determine whether specific pathogens were more likely to be present in co-infections, a network analysis was performed using Spearman’s correlation coefficient via the “igraph” and “bootnet” packages in R software (R Core Team, 2023). The network analysis was performed using only submissions that had results for all six pathogens: *Bordetella bronchiseptica*, *Chlamydia felis*, FCV, FeHV-1, *Mycoplasma* spp., and Influenza A virus.

A univariable ordered logistic regression model was employed to predict the four-tiered outcome of interest. The outcome categories were defined as follows: clinical score 0 represented no infection, score 1 represented upper mild respiratory tract clinical signs, score 2 represented upper severe respiratory tract clinical signs, and score 4 represented lower respiratory tract clinical signs and pneumonia.

The explanatory variables considered in the analysis included age (categorical variable), sex (binary variable), season (binary variable), and six pathogens (binary variables). All samples used in the analysis were obtained from UGA. Supplementary Table S2 displays the frequencies of demographics, temporal characteristics, and the presence and absence of six pathogens. Moreover, this analysis involved the consideration of 10 co-infections consisting of two pathogens as explanatory variables. The frequencies of the presence and absence of these 10 combinations are presented in Supplementary Table S3, with influenza consistently showing a negative result. Furthermore, this analysis included consideration of 10 co-infections involving three pathogens as explanatory variables (Supplementary Table S4). Variables with a proportion of binary results lower than 5% were excluded due to the substantial standard errors produced (Supplementary Tables S2–S4).

## 3 Results

### 3.1 Description of the study population

Between July 2011 and December 2020, 263 clinical specimens were submitted for the feline PCR respiratory panel at laboratory A (Georgia); between July 2015 and May 2019, 251 specimens were submitted for diagnostic testing at laboratory B (New York), and between October 2015 and December 2020, 41 specimens were submitted to laboratory C (Canada). The clinically normal group (control) consisted of 51 cats. Information about clinical signs was missing from 316 cats (Table 1). The entire population (*n* = 555) was composed of 152 males and 159 females; information about sex was unavailable in the submission form for 244 samples. The median age was 2 years old, with a minimum of 1-week-old and a maximum of 22-year-old. The age group was divided into 96 kittens, 89 juniors, 129 adults, and 43 seniors; information about age was unavailable in the submission form of 198 samples. The type of specimens submitted for diagnostics are described in Supplementary Table S1. There was no information about previous shelter residency, vaccination history, breed, indoor and/or outdoor access, number of cats per household, or physical exam data prior to sample submission.

### 3.2 Detection of pathogens by season, sex, age, and clinical condition

**Figure 1:**
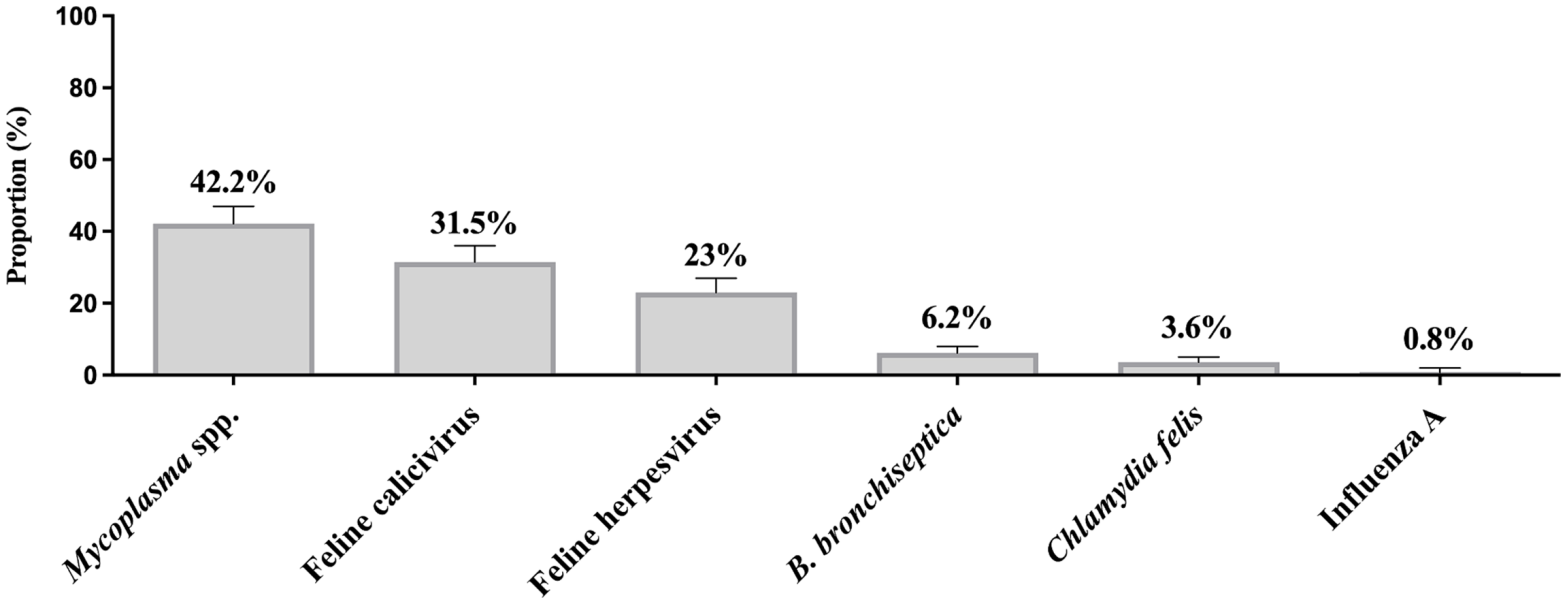
Proportion of feline respiratory pathogens in three veterinary diagnostic laboratories in North America. Clinical specimens were submitted between 2011 and 2020. Total number of samples submitted for *Mycoplasma* testing = 438, Feline calicivirus = 378, Feline herpesvirus (*Felid alphaherpesvirus 1*) = 407, *Bordetella bronchiseptica* = 469, *Chlamydia felis* = 447, and Influenza A virus = 482. Error bars represent 95% confidence intervals.

The proportion of detection of pathogens from all laboratories is illustrated in Figure 1. *Mycoplasma* (42.2%, 184/438), FCV (31.5%, 119/378), and FeHV-1 (23%, 94/407) were the most detected pathogens, followed by *B. bronchiseptica* (6.2%, 29/469), *C. felis* (3.6%, 16/447), and influenza A virus (0.8%, 4/482).

**Figure 2:**
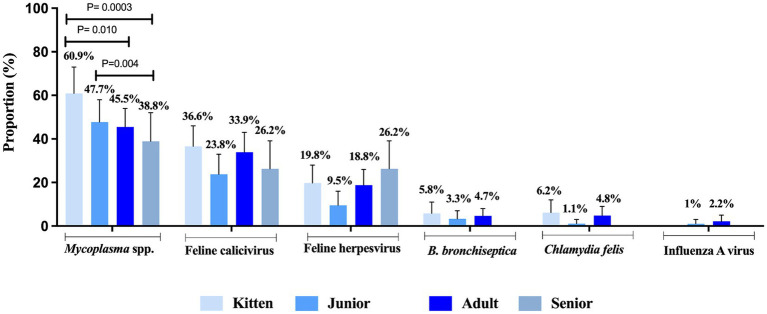
Proportion of feline respiratory pathogens according to seasonality between 2011 and 2020. Two seasons, cold and warm, were determined. Total number of samples submitted for *Mycoplasma* testing: *n* = 218 cold, *n* = 218 warm; Feline calicivirus: *n* = 206 cold, *n* = 170 warm; Feline herpesvirus (*Felid alphaherpesvirus* 1): *n* = 232 cold, *n* = 173 warm; *Bordetella bronchiseptica*: *n* = 226 cold, *n* = 241 warm; *Chlamydia felis*: *n* = 224 cold and *n* = 221 warm; and Influenza A virus: *n* = 247 cold, *n* = 233 warm. Data were analyzed using Fisher’s Exact test due to low cell counts and *p*-values ≤ 0.05 were considered significant. Error bars represent 95% confidence intervals.

The total number of samples received by the three diagnostic laboratories was similar between cold and warm seasons, with a total number of 256 samples received in the cold months and 248 samples received in the summer months. In a dataset of 555 samples, the total counts for *B. bronchiseptica, C. felis*, FCV, FeHV-1, *Mycoplasma* spp., and Influenza A virus were 467, 445, 376, 405, 436, and 480, respectively. However, not all submissions underwent testing for every pathogen. There was a borderline significant difference in the FeHV-1 (*p* = 0.032) and *Mycoplasma* spp. (*p* = 0.052) detection rates according to season, which was more commonly detected in the cold months (Figure 2). There was no significant seasonal difference in the detection rate of FCV, *B. bronchiseptica*, *C. felis,* and influenza A virus.

**Figure 3:**
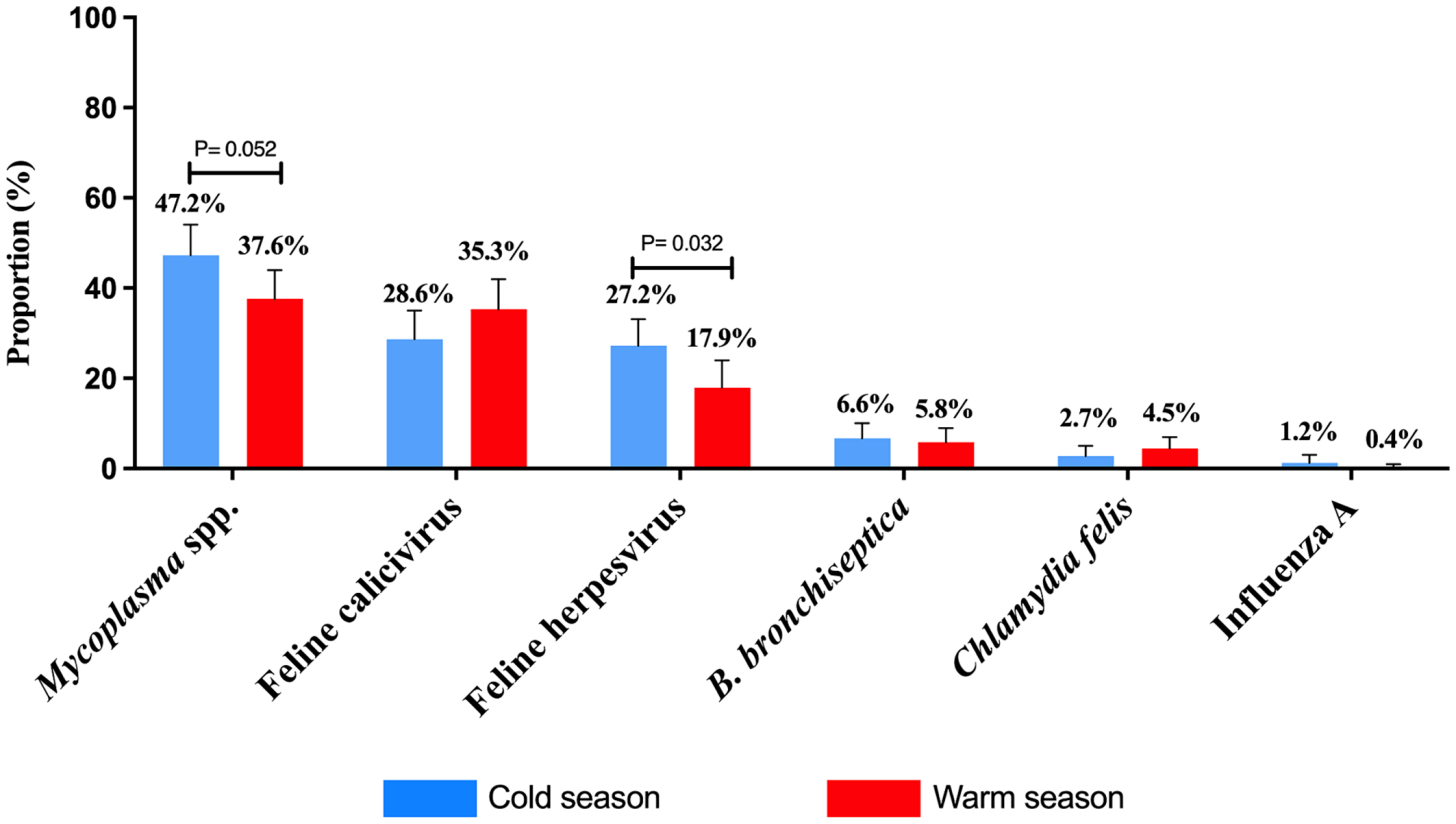
Proportion of feline respiratory pathogens by age. Four categories were evaluated and defined as kitten (1- to <7-month-old), junior (7 month to <3-year-old), adult (3 to ≤11-year-old) and senior (>11-year-old). Data were analyzed using Fisher’s exact test with Holm post-hoc test and p-values ≤0.05 were considered significant. Error bars represent 95% confidence intervals.

FRDC pathogens were present in cats of all age categories without any significant difference among kitten (1- to 6-month-old), junior (7-month to 2-year-old), adult (3- to 10-year-old), or senior (>11-month-old) categories, except *Mycoplasma* which was more commonly identified kittens (60.9%) compared to adult cats (45.5%, *p* = 0.01) and seniors (38.8%, *p* = 0.0003). Additionally, *Mycoplasma* was detected more frequently in juniors (47.7%) compared to seniors (38.8%, *p* = 0.004) (Figure 3).

There were no statistically significant differences in the detection of FRDC pathogens between male and female cats.

**Figure 4:**
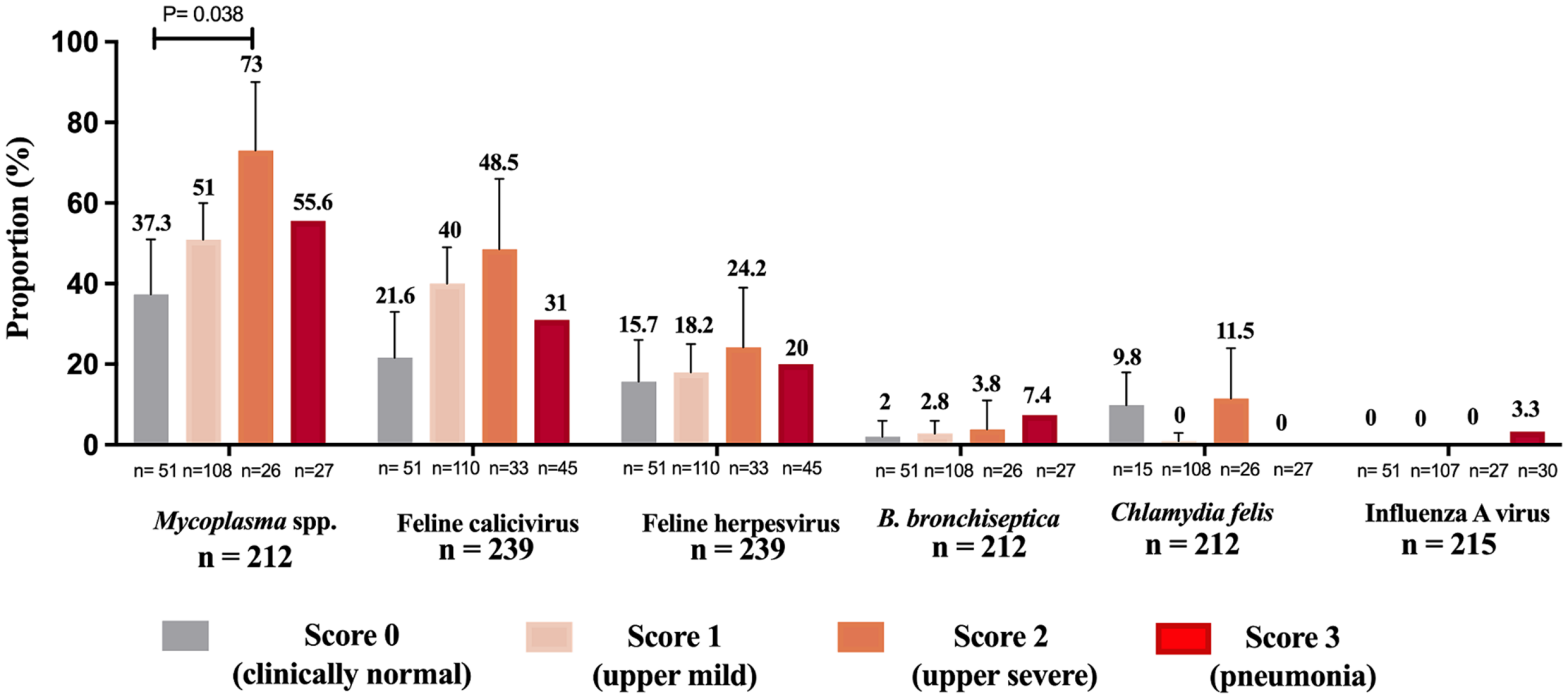
Proportion of feline respiratory pathogens by clinical presentation. Cats were categorized into four clinical score groups: score 0 (clinically normal cats), score 1 upper mild (coughing, sneezing, conjunctivitis, or nasal/eye discharge), score 2 upper severe (coughing, sneezing, conjunctivitis, nasal/eye discharge, in addition to ulcers in the mouth, lethargy, depression, inappetence or fever) and score 3 (pneumonia followed by one or more signs such as dyspnea, lethargy, depression, inappetence or fever). “n” denotes the total counts of positive and negative instances for each pathogen within each clinical score group. The sum of the proportions of cats within each clinical score group across all pathogens may not equal 100%, as a cat can test positive for multiple pathogens or negative for all pathogens, and the presence of one pathogen does not exclude the presence of others. Total n = total number of cats containing a description of clinical signs on the sample submission forms. Data were analyzed using Holm’s *post-hoc* test after Fisher’s exact test and *p*-values ≤ 0.05 were considered significant. Error bars represent 95% confidence intervals.

All assessed pathogens were detected in nasal swabs of clinically normal cats (control group), except for influenza A virus which was not found in the control group (Figure 4). *Mycoplasma* was more frequently detected in cats with clinical signs of severe upper signs (73%) compared to clinically normal (37.3%) cats (*p* = 0.038) (Figure 4). *C. felis* was the only pathogen not found in cats presenting severe lower respiratory disease (pneumonia) (Figure 4).

### 3.3 Association between pathogen and its impact on disease severity

An ordered logistic regression was performed to assess the association between the presence of pathogens and the severity of clinical signs of FRDC. The *Mycoplasma* analysis was based on a sample of 187 observations. Cats with *Mycoplasma* had 1.91 times higher odds of progressing to a more severe clinical score (*p* = 0.021) (Table 2). Specifically, the odds of transitioning from a clinical score of 1 (upper mild respiratory signs) to 2 (upper severe respiratory signs) were 1.91 times greater. Similarly, the odds of moving from a clinical score of 2 (upper severe respiratory signs) to 3 (pneumonia) were also 1.91 times greater in cats infected with *Mycoplasma*. The association between the other tested pathogens and clinical scores was statistically significant.

**Table 2:** The univariable ordered logistic regression models demonstrate associations between predictors (including demographics, temporal characteristics, and pathogens) and the clinical scores of the three-tiered outcome of interest.

Predictor	Value	Number	Odds ratio	Standard error	Z score	*p* value	95% confidence interval
Demographics and temporal characteristics							
Age	Kitten	31	1.78	0.89	1.16	0.245	0.67–4.73
	Junior	48	1.48	0.62	0.94	0.348	0.65–3.35
	Adult	75	1.51	0.58	1.09	0.276	0.72–3.19
	Senior	33	Reference				
Sex	Male	102	0.80	0.22	−1.81	0.417	0.46–1.38
	Female	85	Reference				
Season	Warm	83	1.33	0.37	0.12	0.309	0.77–2.28
	Cold	104	Reference				
Pathogens							
FCV	Present	68	1.72	0.49	1.89	0.059	0.98–3.01
	Absent	119	Reference				
FeHV-1	Present	39	1.80	0.62	1.69	0.090	0.91–3.54
	Absent	148	Reference				
*Mycoplasma* spp.	Present	94	1.91	0.54	2.31	**0.021**	1.10–3.32
	Absent	93	Reference				

Total number of cats assessed = 187.

FCV, Feline calicivirus; FeHV, Felid alphaherpesvirus 1.

Clinical scores are classified as follows: 0 for no clinical signs, 1 for upper respiratory mild disease (coughing, sneezing, conjunctivitis, nasal/eye discharge), 2 for upper respiratory severe disease (ulcers in the mouths, lethargy, depression, inappetence, fever), 3 for lower respiratory disease (pneumonia).

The bold value indicates statistical significance.

### 3.4 Detection of co-infections and its impact on disease severity

**Figure 5:**
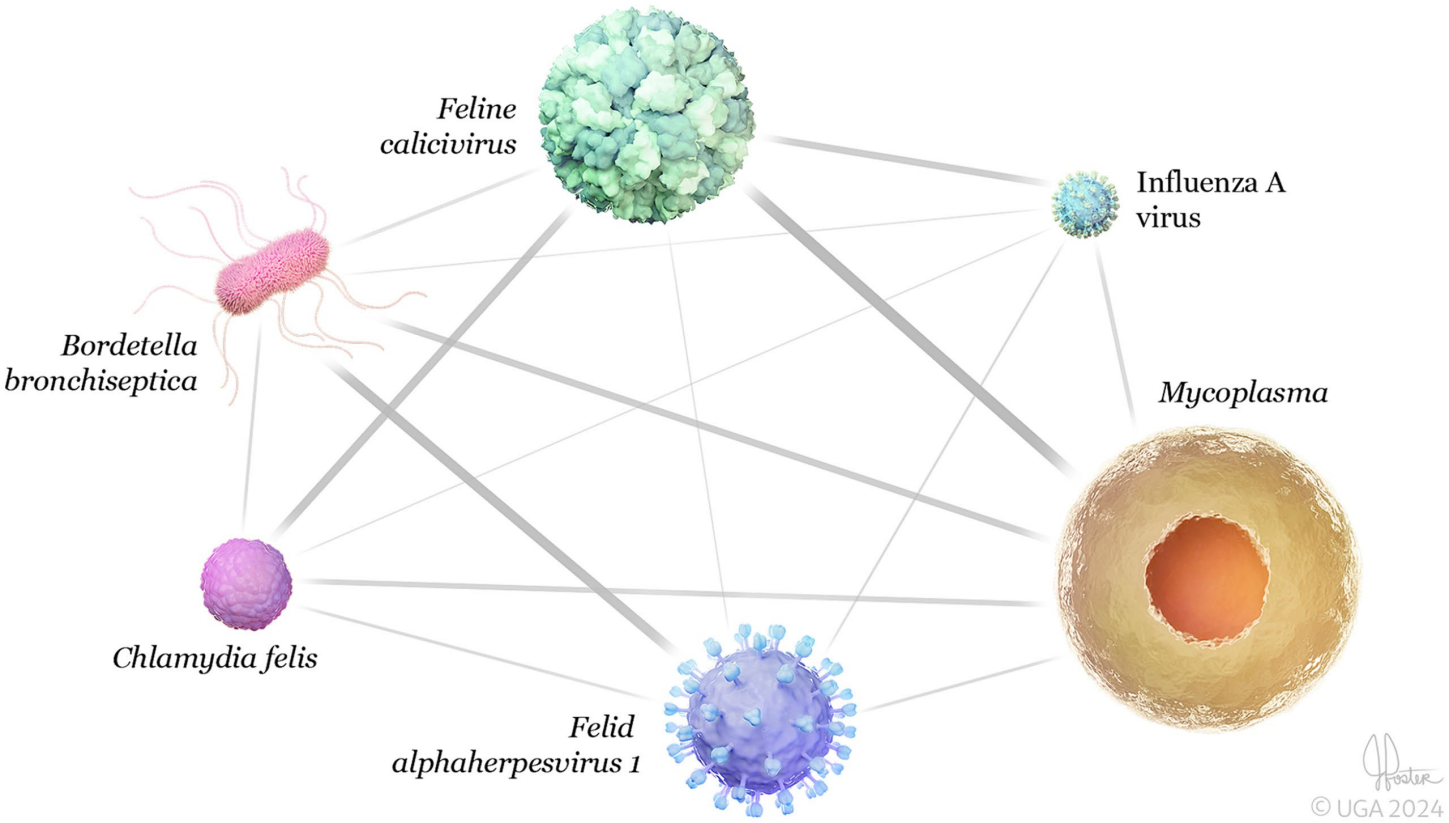
3D network analysis of feline respiratory pathogens detected in the same clinical sample (co-infections) (*n* = 305). The network shows significant connectivity between the strongest co-infections were *Mycoplasma* + *Feline calicivirus* (0.27), followed by *Bordetella bronchipsetica* + *Felid alphaherpesvirus* 1 (0.14), and *Chlamydia felis* + *Feline calicivirus* (0.12). Network analysis was performed using Spearman’s correlation coefficient via the “igraph” and “bootnet” packages in R software (R Core Team, 2023). Error bars represent 95% confidence intervals.

As illustrated in the network analysis, based on submissions that had results for all six pathogens (n = 305), the most common co-infection associations were *Mycoplasma* + FCV followed by *B. bronchiseptica* + FeHV-1 and *C. felis* + FCV (Figure 5).

The co-infection of FCV and *Mycoplasma* was found to be associated with disease severity (*p* = 0.047). Cats co-infected with FCV and *Mycoplasma* had 1.87 times higher odds of progressing to a more severe clinical score. Specifically, the odds of a cat with clinical score of 1 (upper mild respiratory signs) to progress to score 2 (upper severe respiratory signs) were 1.87 times greater. Similarly, the odds of moving from a clinical score of 2 (upper severe respiratory signs) to 3 (pneumonia) were also 1.87 times greater in cats co-infected with FCV and *Mycoplasma*.

Likewise, the co-infection of FeHV-1 and *Mycoplasma* was significantly associated with increased disease severity (*p* = 0.006) (Table 3). Cats co-infected with FeHV-1 and *Mycoplasma* had 3.24 times higher odds of a more severe clinical score. Specifically, the odds of transitioning from a clinical score of 1 (upper mild respiratory signs) to 2 (upper severe respiratory signs) were 3.24 times greater. Similarly, the odds of moving from a clinical score of 2 (upper severe respiratory signs) to 3 (pneumonia) were also 3.24 times greater in cats co-infected with FeHV-1 and *Mycoplasma*.

**Table 3:** The univariable ordered logistic regression model demonstrates associations between predictors (co-infections of two pathogens) and the clinical scores of the four-tiered outcome of interest.

Predictor	Value	Number	Odds ratio	Standard error	Z score	*p* value	95% confidence interval
FCV + FeHV-1	Present	15	1.70	0.85	1.06	0.290	0.64–4.54
	Absent	172					
FCV + *Mycoplasma* spp.	Present	46	1.87	0.59	1.99	**0.047**	1.01–3.47
	Absent	141					
FeHV-1 + *Mycoplasma* spp.	Present	22	3.24	1.38	2.75	**0.006**	1.40–7.47
	Absent	165					

Total number of cats assessed = 187.

FCV, Feline calicivirus; FeHV, Felid alphaherpesvirus 1.

Clinical scores are classified as follows: 0 for no clinical signs, 1 for upper respiratory mild disease (coughing, sneezing, conjunctivitis, nasal/eye discharge), 2 for upper respiratory severe disease (ulcers in the mouths, lethargy, depression, inappetence, fever), 3 for lower respiratory disease (pneumonia).

The bold values indicate statistical significance.

The association between the co-infection of three pathogens (FCV + FeHV-1 + *Mycoplasma* spp.) and disease severity was found to be significant (*p* = 0.038) (Table 4). Cats co-infected with FCV, FeHV-1, and *Mycoplasma* had 3.06 times higher odds of progressing to a more severe clinical score. Specifically, the odds of transitioning from a clinical score of 1 (upper mild respiratory signs) to 2 (upper severe respiratory signs) were 3.06 times greater. Similarly, the odds of moving from a clinical score of 2 (upper severe respiratory signs) to 3 (pneumonia) were also 3.06 times greater in cats co-infected with FCV, FeHV-1, and *Mycoplasma*.

**Table 4:** The univariable ordered logistic regression model demonstrates associations between predictors (co-infections of three pathogens) and the clinical scores of the four-tiered outcome of interest.

Predictor	Value	Number	Odds ratio	Standard error	Z score	*p* value	95% confidence interval
FCV + FeHV-1 + *Mycoplasma*	Present	11	3.06	1.65	2.07	**0.038**	1.06–8.81
	Absent	176					

Total number of cats assessed = 187.

FCV, Feline calicivirus; FeHV, Felid alphaherpesvirus 1.

Clinical scores are classified as follows: 0 for no clinical signs, 1 for upper respiratory mild disease (coughing, sneezing, conjunctivitis, nasal/eye discharge), 2 for upper respiratory severe disease (ulcers in the mouths, lethargy, depression, inappetence, fever), 3 for lower respiratory disease (pneumonia).

The bold value indicates statistical significance.

## 4 Discussion

This study investigated the etiology and epidemiology of feline respiratory pathogens, and the impact of bacterial and viral co-infections in respiratory disease using data generated from the diagnostic work-up of three veterinary diagnostic laboratories in North America. We investigated two main aspects: (i) the proportion of detection of six pathogens according to age, season, sex, and disease severity, (ii) the effect of co-infections on the severity of clinical signs. *Mycoplasma*, FCV, and FeHV-1 were the most detected pathogens across the tree different laboratories. Cats positive for *Mycoplasma* had higher odds of progressing to a more severe clinical score. Further, cats simultaneously co-infected with *Mycoplasma*, FCV, and FeHV-1 were more likely to present with more severe clinical signs. All pathogens were detected in the clinically normal group of cats (control), highlighting the challenge of interpreting positive laboratory testing results. Age, sex, and season had a minor impact on the occurrence of pathogens.

The detection of pathogens in the control cats agrees with previous studies. Respiratory pathogens such as FCV, FeHV-1, *Mycoplasma*, *C. felis* and *B. bronchiseptica* can be present in clinically normal cats (Bannasch and Foley, 2005; Lee-Fowler, 2014; Berger et al., 2015; Fernandez et al., 2017; Nguyen et al., 2019). Here, we detected all these pathogens in the control group, but with lower detection than in the diseased cats (Figure 4). These results highlight that laboratory testing needs to be carefully interpreted, and positive test results might only indicate disease if the cat has clinical signs or pathological lesions of respiratory infection.

Our study revealed a significant association between the presence of *Mycoplasma* and disease severity (Figure 4). Whether this pathogen acted as a primary agent responsible for the clinical signs or acted secondarily to the disease could not be established in this study. *Mycoplasma* is well documented as a primary or opportunistic pathogen in cats; however, it is also a known commensal of the feline upper respiratory tract (Lee-Fowler, 2014). Regardless of whether *Mycoplasma* acts as a primary or an opportunistic pathogen, a positive laboratory result associated with characteristic clinical signs of FRDC suggests that it might be associated with the causation and/or exacerbation of the clinical presentation. FCV was the second most prevalent agent in this study, followed by FeHV-1 (Figure 1). FCV disease is characterized by oral ulcerations, sneezing, nasal discharge, and, less commonly, pneumonia (Litster, 2021; Berger et al., 2015). FCV is commonly found in clinically normal cats, and as expected, we detected FCV in 21.6% in the control group (Figure 4). Despite being less commonly associated with lower respiratory infection, we detected a high proportion of FCV in cats with clinical score 3 (31%, Figure 4). Likewise, FeHV-1 was detected in a high proportion of cats with pneumonia (20%, Figure 4). We assumed that most of the cats in our study received the complete course of core vaccines, which includes immunization against FeHV-1 and FCV. The high proportion of FeHV-1 and FCV-positive cats observed in this study might result from vaccination failure, incomplete vaccination course, or infection prior to vaccination.

The most common co-infections simultaneously detected in the same clinical sample were *Mycoplasma +* FCV and *Mycoplasma* + FCV + FeHV-1. In a previous study, cats infected with *M. felis* were significantly more likely to be co-infected with FCV, FHV, *C. felis*, or *B. bronchiseptica* (McManus et al., 2014). We further investigated whether co-infections were significantly associated with disease severity using the univariable regression analysis. Based on our results, FeHV-1 only impacted disease severity in simultaneous co-infection with *Mycoplasma* and FCV. Co-infections with FCV and FeHV-1 have often been found to occur concurrently (Schulz et al., 2015), and cats co-infected with FCV and FeHV-1 were reported to have a high risk of developing clinical signs (Chandler et al., 2008). In another study, FeHV-1 infection alone was associated with increased severity of respiratory and ocular signs in cats from Spain (Fernandez et al., 2017). Other co-infections found in our dataset were FCV and *C. felis*, and FeHV-1 and *B. bronchiseptica*. Co-infections with FeHV-1 and *B. bronchiseptica* might be common in cats with upper respiratory disease (Litster et al., 2015). Here, we highlight the relevance of respiratory diagnostic panels to screen for a wide range of pathogens simultaneously associated with FRDC. Recent studies have developed multiplex PCRs for several FRDC pathogens, including quantitative multiplex PCR (Thieulent et al., 2024) and conventional PCR assays (Xiao et al., 2022). Such panels and multiplex PCRs provide knowledge for timely diagnosis and direct therapeutic interventions against bacterial and viral infections. Still, interpretation remains challenging based on the presence of these pathogens in healthy cats.

This study has limitations. Finding an ideal control group of clinically normal cats for a retrospective and diagnostic-based study was challenging. Our approach was to include necropsied animals to ensure the absence of lesions in the respiratory tract as previously reported by our group (Maboni et al., 2019). We selected the necropsied cats based on the absence of clinical history and macroscopic and histologic findings of respiratory disease. Another limitation was the lack of information about whether clinical samples were from cats vaccinated against the investigated pathogens; therefore, we do not know if PCR-positive results were due to vaccine strains. However, in a shelter-based study, FeHV-1 PCR results were not affected by recent vaccination (McManus et al., 2014). Further, clinical history was unavailable in the dataset from laboratory B. Information about immunosuppressive viruses such as Feline leukemia Virus, Feline Immunodeficiency Virus, and Feline Infectious Peritonitis Virus was unavailable in this study since the feline respiratory testing panels offered by the laboratories did not include such viruses. Further studies are warranted to investigate the role of immunosuppressive viruses in co-infections, considering they may trigger respiratory infectious diseases. Another methodological limitation to be considered when interpreting the results is that different targeted pathogens were detected using different methods (qPCR and virus isolation) across the three laboratories involved. Even within the qPCR method, different assays were employed. We acknowledge that these methodological discrepancies could impact the strength of our conclusions. Future studies should aim to use standardized techniques and assays across all participating laboratories to ensure more comparable results. Despite these limitations, our study provides valuable insights into the prevalence and co-occurrence of these pathogens, serving as a foundation for further research in this area.

## 5 Conclusion

This study provides new insights into the current knowledge of the occurrence of pathogens and the potential role of co-infections in feline respiratory disease in North America. Key findings were that *Mycoplasma*, FCV, and FeHV-1 were the most detected pathogens across the tree different laboratories. Further, co-infections, especially with *Mycoplasma*, FeHV-1 and FCV, were associated with severe clinical presentation. Other common co-infections found in our dataset were *B. bronchiseptica* + FeHV-1 and *Mycoplasma* + *B. bronchiseptica.* All pathogens were detected in the control group of cats, highlighting the challenge of interpreting positive laboratory testing results and the fact that positive test results might only indicate disease if the cat presents with clinical signs or pathological lesions of respiratory infection. This study may contribute to developing or refining preventative therapeutics at a time when the efficacy of antimicrobial treatments is under pressure. Further, the information presented here underscores the importance of comprehensive diagnostic panels to facilitate the selection of appropriate clinical management and control measures.

## Data Availability

The original contributions presented in the study are included in the article/Supplementary material, further inquiries can be directed to the corresponding author.
