# Supplementary material for: Feline respiratory disease complex: insights into the role of viral and bacterial co-infections
Source: Front Microbiol. 2024 Sep 3;15:1455453. doi: 10.3389/fmicb.2024.1455453 (PMC11405337; doi:10.3389/fmicb.2024.1455453)
Supplement: Supplementary file 1 [file Data_Sheet_1.pdf]

Supplementary Table 1. Specimen type and number of specimens.

Specimen type	Number of specimens
Conjunctiva and nasal swab	6
Conjunctiva and oropharyngeal swab	54
Conjunctival, nasal, and oropharyngeal swab	16
Eye swab	25
Lung	51
Nasal swab	248
Oropharyngeal swab	105
Transtracheal wash	12
Unknown	38
**Total**	**555**

Supplementary Table 2. Frequency of variables including demographics, temporal characteristics, and six pathogens.

Variables	Frequency	Percent
**Demographic and temporal characteristics**		
Age of cats		
Kitten	31	16.6
Junior	48	25.7
Adult	75	40.1
Senior	33	17.6
Sex of cats		
Female	85	45.5
Male	102	54.5
Season		
Cold	104	55.6
Warm	83	44.4
**Six pathogens**		
*B. bronchiseptica**		
Absent	182	97.3
Present	5	2.7
*Chlamydia felis**		
Absent	179	95.7
Present	8	4.3
FCV		
Absent	119	63.6
Present	68	36.4
FeHV		
Absent	148	79.1
Present	39	20.9
Influenza*		
Absent	187	100.0
Present	0	0.0
Mycoplasma spp.		
Absent	93	49.7
Present	94	50.3

FCV: *Feline calicivirus*, FeHV: *Felid alphaherpesvirus 1*
Variables are indicated by an asterisk (*) when the proportion of the binary results is lower than 5%.
Number of tested cats: 187.

Supplementary Table 3. Frequency of absence and presence of co-infection of two pathogens in cats. Cats with zero or one pathogen are classified as absence, while cats testing positive for two pathogens are classified as presence.

**Variables**	**Frequency**	**Percent**
B. bronchiseptica + *Chlamydia felis**		
Absent	186	99.5
Present	1	0.5
B. bronchiseptica + FCV*		
Absent	185	98.9
Present	2	1.1
B. bronchiseptica + FeHV*		
Absent	185	98.9
Present	2	1.1
B. bronchiseptica + *Mycoplasma* spp.*		
Absent	183	97.9
Present	4	2.1
Chlamydia felis + FCV*		
Absent	183	97.9
Present	4	2.1
Chlamydia felis + FeHV*		
Absent	184	98.4
Present	3	1.6
Chlamydia felis + *Mycoplasma* spp.*		
Absent	182	97.3
Present	5	2.7
FCV + FeHV		
Absent	172	92.0
Present	15	8.0
FCV + *Mycoplasma* spp.		
Absent	141	75.4
Present	46	24.6
FeHV + *Mycoplasma* spp.		
Absent	165	88.2
Present	22	11.8

B. bronchiseptica: Bordetella bronchiseptica, FCV: Feline calicivirus, FeHV: Felid alphaherpesvirus 1.
Variables are indicated by an asterisk (*) when the proportion of the binary results is lower than 5%.
Number of tested cats: 187.

Supplementary Table 4. Frequency of absence and presence of co-infection of three pathogens in cats. Cats with zero, one, or two pathogens are classified as absence, while cats testing positive for three pathogens are classified as presence.

Variables	Frequency	Percent
B. bronchiseptica + *Chlamydia felis* + FCV*		
Absent	187	100.0
Present	0	0.0
B. bronchiseptica + *Chlamydia felis* + FeHV-1*		
Absent	186	99.5
Present	1	0.5
B. bronchiseptica + *Chlamydia felis* + *Mycoplasma* spp.*		
Absent	187	100.0
Present	0	0.0
B. bronchiseptica + FCV + FeHV-1*		
Absent	185	98.9
Present	2	1.1
B. bronchiseptica + FCV + *Mycoplasma* spp.*		
Absent	186	99.5
Present	1	0.5
B. bronchiseptica + FeHV-1 + *Mycoplasma* spp.*		
Absent	186	99.5
Present	1	0.5
Chlamydia felis + FCV + FeHV-1*		
Absent	185	98.9
Present	2	1.1
Chlamydia felis + FCV + *Mycoplasma* spp.*		
Absent	185	98.9
Present	2	1.1
Chlamydia felis + FeHV-1 + *Mycoplasma* spp.*		
Absent	187	100.0
Present	0	0.0
FCV + FeHV-1 + *Mycoplasma* spp.		
Absent	176	94.1
Present	11	5.9

B. bronchiseptica: Bordetella bronchiseptica, FCV: *Feline calicivirus*, FeHV-1: *Felid alphaherpesvirus 1*
Variables are indicated by an asterisk (*) when the proportion of the binary results is lower than 5%.
Number of tested cats: 187.
